# Dose‐Dependent Effects of Biochar on Soil Revealed by Fast Field‐Cycling (FFC) NMR: From Molecular Water Dynamics to Soil Functionality

**DOI:** 10.1002/mrc.70077

**Published:** 2025-12-31

**Authors:** Calogero Librici, Paola Bambina, Ettore Madonia, Veronica Ciaramitaro, Delia Francesca Chillura Martino, Paolo Lo Meo, Pellegrino Conte

**Affiliations:** ^1^ Dipartimento di Scienze Agrarie, Alimentari e Forestali (SAAF) Università Degli Studi di Palermo Palermo Italy; ^2^ Dipartimento di Scienze e Tecnologie Biologiche Chimiche e Farmaceutiche (STeBiCeF) Università Degli Studi di Palermo Palermo Italy

**Keywords:** biochar, bulk density, clay soil, dose–response curve, electrical conductivity, fast‐field‐cycling NMR, FT‐IR spectroscopy, soil physicochemical properties, water activity, water‐holding capacity

## Abstract

Biochar is a multifunctional soil amendment that improves soil structure, enhances water‐holding capacity, and contributes to carbon sequestration. However, the dose–response relationship between biochar addition and soil behavior remains underexplored, particularly at high application rates. In this study, fifteen soil–biochar mixtures were prepared with biochar mass fractions from 0 to 1 (*f*_BC_ = 0–1) to evaluate in detail the changes induced in a Sicilian clay soil. The mixtures were investigated for pH, electrical conductivity, bulk density, water‐holding capacity, and water activity (Aw). Biochar addition caused pronounced increases in alkalinity, porosity, and water retention, following nonlinear dose–response trends with clear thresholds beyond *f*_BC_ ≈ 0.3–0.5. FT‐IR spectroscopy revealed the progressive appearance of oxygenated and aromatic functional groups, accompanied by a reduction in signals from adsorbed water and native soil polar groups. Fast Field‐Cycling NMR relaxometry provided molecular‐scale insight into soil–water interactions. At high biochar contents, water proton *T*_1_ relaxation times were markedly lengthened, indicating a reduced overall efficiency of surface‐driven relaxation. Correlation‐time (*τ*_c_) analysis further revealed the emergence of water populations with longer correlation times and a redistribution of relaxation pathways toward outer‐sphere dominated mechanisms. Overall, the results indicate that biochar improves soil water retention not by strong surface adsorption but through effective pore‐space storage, keeping water available for biological use. The combined spectroscopic and relaxometric approach establishes a direct link between molecular‐level water dynamics and macroscopic soil properties, highlighting the value of FFC‐NMR as a powerful tool for studying natural porous systems.

## Graphical abstract

Dose–response of biochar in a clay soil: fifteen mixtures (*f*_BC_ 0–1) reveal nonlinear thresholds (pH/EC) and sharp WHC gains near *f*_BC_ ≈ 0.3–0.5. Coupled FT‐IR and FFC‐NMR (0.01–10 MHz; ModelFree *τ*_c_ distributions) link water dynamics to soil functionality. Systematic T_1_ lengthening indicates less surface‐constrained water and a pore‐storage‐dominated mechanism. Implication: moderate application rates deliver maximal, sustainable benefits.

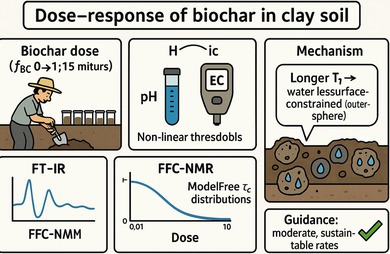

## 1 Introduction

Soil is a highly complex system of paramount importance because of its direct role in food and fiber production. In recent years, soil loss driven by intensive human activities has become one of the major environmental challenges faced by humankind [1, 2, 3, 4, 5]. Several strategies have been developed to either prevent soil degradation or restore damaged soils to their native conditions. For example, reforestation with native vegetation has been shown to be effective in rehabilitating severely degraded Brazilian soils [6, 7]. Likewise, good agricultural practices such as no‐tillage, cover crops, and the sustainable use of agrochemicals have been proposed to maintain fertility in stressed agricultural soils [8, 9, 10, 11]. In addition, new materials—including biochar (bc)—are being studied as amendments to improve soil quality [12, 13, 14]. Biochar is a carbon‐rich material obtained through the thermal degradation of biomass under oxygen‐starved conditions (i.e., pyrolysis) [15]. A series of structural rearrangements occurs, leading to the retention of carbon in a porous matrix with unique physical and chemical properties. Its high chemical stability is particularly remarkable, especially in comparison with the organic matter typically present in soils [12]. Because of this high stability, biochar is considered an effective means of temporarily sequestering CO_2_ from the global carbon cycle, thereby helping to reduce its atmospheric concentrations [12]. Beyond CO_2_ sequestration, the incorporation of biochar into soils has also been shown to mitigate emissions of other greenhouse gases, such as nitrous oxide, while simultaneously enhancing nitrogen availability for plant nutrition [16, 17, 18, 19]. In general, the application of biochar—and its modified forms [20]—improves soil quality through multiple mechanisms—i.e., by providing a continuous source of plant nutrients [21, 22, 23, 24], by stimulating microbial activity [25, 26], by increasing soil organic matter content [25, 27, 28], by altering soil structure [11, 21, 29, 30, 31], by enhancing water infiltration [32], and by reducing soil loss due to erosion [33]. Finally, biochar is also known to buffer soil pH [12, 21]. However, it should also be noted that some studies have reported no significant effects of biochar on soil properties [34, 35, 36], while others have even documented adverse outcomes following its application [11, 37, 38, 39, 40, 41]. On the one hand, Peng and co‐workers [35] observed that biochar had no effect on soil erosion because it became incorporated into the clay soil matrix. Schulz et al. [36], conversely, reported that a no‐effect level of at least 3 t ha^−1^ could be identified—in terms of plant growth, total organic carbon, total nitrogen, pH, and nutrient availability—when biochar was applied as a co‐composted material on oat ( *Avena sativa* L.). On the other hand, Li et al. [38] showed that biochar reduced soil loss, but that the effect was reversed in tilled soils; Kumar et al. [37] reported that the hydrophobic properties of biochar exacerbated soil erosion; cautioned that applying high doses of biochar (i.e., up to 8%, w/w^−1^, that is ≈ 220 t ha^−1^) on sloping soils may actually increase the risk of erosion. It is worth noting that the effects of biochar on soil properties depend on several factors. In particular, the amount of biochar applied [11], the type of soil [34, 40], the feedstock used, and the pyrolysis conditions [12, 39, 40] play major roles. However, regarding the amount of biochar applied to soils (hereafter referred to as *f*_BC_), it is evident that in much of the existing literature the choice of *f*_BC_ values has often been arbitrary. For instance, Chan et al. [42] used arbitrarily selected doses of a biochar to examine its impact on fertility. Similarly, Spokas et al. [19] investigated greenhouse gas emissions from soils treated with biochar at arbitrarily chosen levels. Schmidt et al. [24] reported that an arbitrarily selected dose of biochar resulted in a fourfold increase in pumpkin yield. Baiamonte et al. [30] studied the hydraulic properties of a sandy soil amended with three biochar rates chosen on the basis of previous studies by Spokas and Chan [19, 42]. Abrol et al. [32] applied four different biochar rates, again arbitrarily selected, to two soils in order to evaluate the hydrological implications. Peng et al. [35] considered aggregation and erosion in a hillslope soil amended with only one biochar rate. Li et al. [38] evaluated the effect of a single biochar dose on erosion and nutrient loss at two experimental sites. Kumar et al. [37] assessed the erodibility of compacted biochar‐amended soils using only two arbitrarily chosen biochar doses. Finally, Ding et al. [27] reported that applying four randomly selected biochar rates significantly increased soil organic carbon under conservation tillage in a 11‐years field experiments.

Traditional soil analyses (e.g., pH, EC, bulk density, and WHC) provide essential information on the macroscopic changes induced by biochar, but they cannot directly capture how water molecules interact with soil–biochar matrices at the molecular scale. Fast Field‐Cycling NMR (FFC‐NMR) relaxometry provides field‐dependent longitudinal relaxation rates *R*_1_(*ν*_L_), which are sensitive to water molecules at solid surfaces and within pore networks of heterogeneous media, thus offering a complementary molecular‐scale view of soil–biochar systems [43]. In parallel, low‐field ^1^H NMR approaches at fixed field, including *T*_1_/*T*_2_ relaxometry and diffusion–difference NMR (DDIF), have been applied to investigate biochar porosity, ageing and water–biochar interactions, and to relate these features to contaminant retention [44, 45]. Together, these studies demonstrate the potential of low‐field NMR to characterize biochar structure and its interaction with water; however, they typically focus on individual biochars or on limited soil–biochar systems and do not explore systematic dose–response series of soil–biochar mixtures. In the present work, we use FFC‐NMR relaxometry, combined with FT‐IR spectroscopy and standard soil measurements, to follow how water dynamics and surface chemistry evolve along a wide gradient of biochar mass fraction in a clay agricultural soil. The existing literature on biochar application rates may not cover all relevant studies, yet it consistently highlights a lack of coherent rationale in selecting dosage levels. To address this gap, the present study aims at providing a systematic evaluation of a wide range of biochar doses—broader than those usually reported in the literature—on the physicochemical properties of a clay soil from Sicily (Italy). Specifically, 15 different *f*_BC_ values were tested, ranging from 0 (soil only) to 1 (biochar only), corresponding to application rates between ≃ 6 and 6 × 10^3^ t ha^−1^. Although the highest values are clearly unrealistic under field conditions, they offer valuable insight into how soils respond when biochar is applied at inappropriate or excessive levels.

## 2 Materials and Methods

### 2.1 Soil Sample

The soil used in this study was sampled from the surface layer (0–5 cm) at the Sparacia experimental station of the University of Palermo. The experimental station, located in western Sicily, 100 km south of Palermo (37°38′12.48″ N, 13°45′57.71″ E), is characterized by a semi‐arid Mediterranean climate with mild and wet winters and hot and dry summers [46]. The soil is a *Vertic Haploxerept* [47] with a clayey texture, according to USDA classification (clay = 62.1%, silt = 21.8%, and sand = 16.1%) and a negligible gravel content. The soil appears massive in winter when it is wet and fully swollen but develops a polygonal pattern of surface shrinkage cracks in late spring or early summer as the soil dries.

Soil bulk density ( D) was measured by using a 1000 cm^3^ cylinder via the relationship (1):

(1) $$D=\frac{M}{V}\;\left(g\;{\mathrm{cm}}^{-3}\right)$$

where M is the weight of the sample (g), and V is the volume of the container. $D_{\text{soil}}$ resulted 1.325 ± 0.002 g cm^−3^. The standard deviation was obtained by replicating trice the bulk density measurements.

### 2.2 Biochar Sample

The biochar sample used in this study was produced from poplar wood chips (*Populus* spp. L.) harvested from a short rotation forestry located in the Po Valley (Gadesco Pieve Delmona, latitude 45°10′13′′ N, longitude 10°06′01′). The forestry was 5 years old at the time of harvesting [48]. The main chemical–physical bc characteristics, as well as the bc production procedure, have been reported elsewhere [48]. Relationship (1) was used to measure BC bulk density, which was 0.173 ± 0.001 g cm^−3^. The standard deviation was calculated from three replicate measurements.

### 2.3 Soil‐Biochar Mixture Preparation

Soil‐biochar mixtures were prepared by mixing an amount of soil ( $P_{s}$ in g) with one of biochar ( $P_{\mathrm{BC}}$ in g) in order to obtain biochar fraction ( $f_{\mathrm{BC}}$) according to Baiamonte et al. [30]:

(2) $$f_{\mathrm{BC}}=\frac{P_{\mathrm{BC}}}{P_{s}+P_{\mathrm{BC}}}$$

Table 1 reports the different $f_{\mathrm{BC}}$ values used in the present study. By assuming a soil depth of 5 cm and considering the soil density reported above, the selected $f_{\mathrm{BC}}s$ correspond to a tonnage per hectare (t ha^−1^) ranging from a minimum of 6.6 to a maximum of 5850 t ha^−1^ (Table 1). As already reported in the Introduction, we are aware that the largest tonnages per hectare are impractical on the field. However, we also studied extreme conditions to gain a complete and clear understanding of what happens when an inappropriate amount of biochar is used as a soil amendment. Prior to all the experiments, all samples were sieved through a 2 mm mesh and then dried in an oven at 60°C for 24 h.

**TABLE 1:** Fractions of biochar used in the present study and corresponding amount in t ha^−1^.

Fraction of biochar ( $f_{\mathrm{BC}}$)	t ha^−1^	Fraction of biochar ( $f_{\mathrm{BC}}$)	t ha^−1^
0	0	0.4	433
0.01	6.6	0.5	650
0.03	20	0.6	975
0.05	34	0.7	1517
0.07	42	0.8	2600
0.1	72	0.9	5850
0.2	162	1	//
0.3	279		

### 2.4 pH Measurements

The pH of all the samples was measured using a glass pH meter with a precision of 0.1 pH units (FiveEasy, Mettler ToledoSpa, Milan, Italia), using a 1:5 sample‐to‐water ratio (deionized water). Prior to measurement, the sample was shaken for 24 h to ensure exchange of all the ions, then filtered to obtain the filtrate. The pH meter probe was inserted into the filtrate to a depth of about 5 cm and the pH reading was recorded after the probe had reached reading stability, usually within 1 min. To minimize cross‐contamination between samples, the pH meter probe was carefully cleaned with deionized water and dried between measurements.

### 2.5 Electrical Conductivity (EC)

The EC of the soil was measured using a conductivity meter with a precision of 0.1 mS cm^−1^ (HI5321 Hanna Instruments Italia Srl, Padua, Italia) using the same procedure described for the pH measurement.

### 2.6 Bulk Density Measurements

The bulk densities of all the mixtures were calculated according to Adams [49]:

(3) $$p_{b}=\frac{p_{1}p_{2}}{p_{2}f_{\mathrm{BC}}+\left(1-f_{\mathrm{BC}}\right)p_{1}}$$

In (3), $p_{b}$ is the bulk density of the mixture in g cm^−3^; $f_{\mathrm{BC}}$ is the biochar fraction as reported in Table 1; $p_{1}$ is the BC bulk density, and $p_{2}$ is the bulk density of the soil. All the bulk densities are listed in Table 2.

**TABLE 2:** Bulk densities of the samples analyzed in the present study. With the exception of $f_{\mathrm{BC}}=0$, and $f_{\mathrm{BC}}=1$ for which the bulk densities were measured trice, the bulk densities and corresponding errors for the samples with $f_{\mathrm{BC}}$ ranging between 0 and 1 were calculated according to relationship (3), and the theory of errors.

Fraction of biochar ( $f_{\mathrm{BC}}$)	Bulk density (g cm^−3^)	Fraction of biochar ( $f_{\mathrm{BC}}$)	Bulk density (g cm^−3^)
0	1.325 ± 0.002	0.4	0.36 ± 0.01
0.01	1.24 ± 0.04	0.5	0.31 ± 0.01
0.03	1.10 ± 0.04	0.6	0.265 ± 0.009
0.05	0.99 ± 0.03	0.7	0.234 ± 0.008
0.07	0.90 ± 0.03	0.8	0.209 ± 0.007
0.1	0.80 ± 0.03	0.9	0.189 ± 0.007
0.2	0.57 ± 0.02	1	0.173 ± 0.001
0.3	0.44 ± 0.02		

### 2.7 Structural and Surface Properties

The structural and surface properties of the samples were determined with a Quantachrome Nova 2200 Multi‐Station High Speed gas sorption analyzer. Prior to analysis, the samples were degassed at 200°C for 2 h to remove any moisture and other gases eventually adsorbed on the surface. Adsorption and desorption curves were acquired by using nitrogen gas. Nitrogen adsorption–desorption isotherms were then collected. For Brunauer–Emmett–Teller (BET) surface‐area calculations, data in the relative pressure range 0.15–0.30 p/p_0_ were used, corresponding to the linear region of the BET plot for these materials and consistent with recommended practice for soils and biochars [50]. The Barrett–Joyner–Halenda (BJH) method was applied to the desorption branch to obtain pore‐size distributions and pore volumes.

### 2.8 Water Holding Capacity (WHC)

The water holding capacity (WHC %) for each sample was determined using a 10 mL plastic syringe following the method described by Di Vincenzo et al. [51]. Specifically, a small piece of hydrophilic cotton was placed at the tip of the syringe. Then, the syringe was filled with an amount of sample ranging between ca. 150–500 mg. The filled syringe was placed in a 100 mL beaker containing ≈80 mL distilled water to allow capillary rise of water. After the system reached equilibrium (in about 2 h), the amount of retained water was measured by weight according to the relationship. (4):

(4) $$\mathrm{WHC}\%=\left(\frac{P_{f}-P_{t}}{P_{i}-P_{t}}\right)*100$$

where *P*_*t*_, *P*_*i*_, and *P*_*f*_ are the weights of the empty syringe, the syringe with the dry material, and the syringe with the completed absorption, respectively. All the WHC % are reported in Table 4. For each material, the measurement protocol was repeated over four consecutive wetting–drying cycles; the values reported correspond to the mean of the last 3 cycles, when WHC values had stabilized, in order to ensure measurement reproducibility

### 2.9 Water Activity

To determine the water activity ( $A_{W}$) of each sample, an activity water meter (HygroPalm‐23, Rotronic, Basserdorf, Germany) was used. The measurements were performed in triplicate to ensure the reliability of the results.

The $A_{W}$ value was measured according to the relationship (5):

(5) $$A_{W}=\frac{P}{P_{0}}$$

where *P* represents the vapor pressure of water in the analyzed sample, and *P*_0_ is the vapor pressure of pure water at the same temperature. $A_{W}$ ranges from 0 to 1, where 0 indicates a completely bound water and 1 represents the free unbound water.

### 2.10 Infrared Spectroscopy (FT‐IR)

To determine the functional groups present in the soil and biochar mixtures, Fourier transform attenuated total reflectance infrared spectra (ATR‐FT‐IR) were acquired in the range of 400–4000 cm^−1^ by a PerkinElmer FT‐IR spectrometer Spectrum Two. A total of 64 scans were acquired for each spectrum recorded at a resolution of 4 cm^−1^. Spectra were acquired and processed using the PerkinElmer software. During the spectrum acquisition, the biochar sample was properly positioned in the measurement cell of the spectrometer, ensuring a uniform distribution of the sample on the measurement surface.

### 2.11 ^1^H Fast Field Cycling (FFC) NMR Relaxometry Analyses

Soil–biochar mixtures (1.0 g) were adjusted to their WHC with Milli‐Q water, transferred to 10 mm NMR tubes, sealed and equilibrated overnight at room temperature. FFC‐NMR measurements were carried out on a Stelar SmarTracer bench‐top relaxometer (Stelar, Mede, PV, Italy). For each mixture, proton longitudinal relaxation times *T*_1_ were acquired over a Larmor‐frequency range of 0.01–10 MHz using pre‐polarized (PP) and non‐polarized (NP) sequences at a fixed acquisition field of 7.2 MHz, with a PP/NP crossover set at 3 MHz. Magnetization decays (PP) and recoveries (NP) were fitted by nonlinear least squares to a single stretched‐exponential function, yielding one effective *T*_1_ value per sample and frequency. Relaxation rates *R*_1_(*ν*_L_) = 1/*T*_1_ were then calculated to obtain nuclear magnetic relaxation dispersion (NMRD) profiles [52, 53]. The complete set of *T*_1_ values for all mixtures and frequencies is reported in Table S1. NMRD curves were analyzed with the ModelFree approach to extract distributions of correlation times *τ*_c_ [54, 55]; additional details on pulse sequences, fitting procedures and diagnostics are provided in Methods S1 in the Supporting Information.

### 2.12 Statistical and Modelling Analysis

Dose–response curves for pH, EC, bulk density, BET surface area, pore volume, and water‐holding capacity (WHC) were fitted with parametric models as follows: an exponential approach‐to‐plateau for pH and bulk density, and a three‐parameter logistic (“slogistic1”) for EC, BET, pore volume, and WHC. Let *x* denote the biochar mass fraction $f_{\mathrm{BC}}$ (w/w) and *y* the response variable.

- **Exponential (approach to plateau)** for pH and bulk density:

(6) yx=y0+Ae−xk

- **Logistic 3‐parameter (“slogistic1”)** for EC, BET, pore volume, and WHC:

(7) $$y\left(x\right)=\frac{a}{1+e^{-k\left(x-x_{c}\right)}}$$

These functional forms were chosen because the experimental dose–response curves show clear saturation, with rapid changes at low *f*_BC_ and finite plateaus at higher doses; in addition, some variables (EC, BET surface area, pore volume, and WHC) exhibit a sigmoidal trend with an identifiable inflection region. The exponential approach‐to‐plateau model was therefore used for monotonic responses tending to a single asymptote (pH and bulk density), whereas the three‐parameter logistic model was applied to sigmoidal responses. The fitted curves provide a compact quantitative description of the nonlinear trends and were used to estimate indicators such as thresholds, inflection doses, and asymptotic values, which help to identify ranges where additional biochar inputs yield diminishing returns.

## 3 Results

### 3.1 Evaluation of pH and EC

**FIGURE 1:**
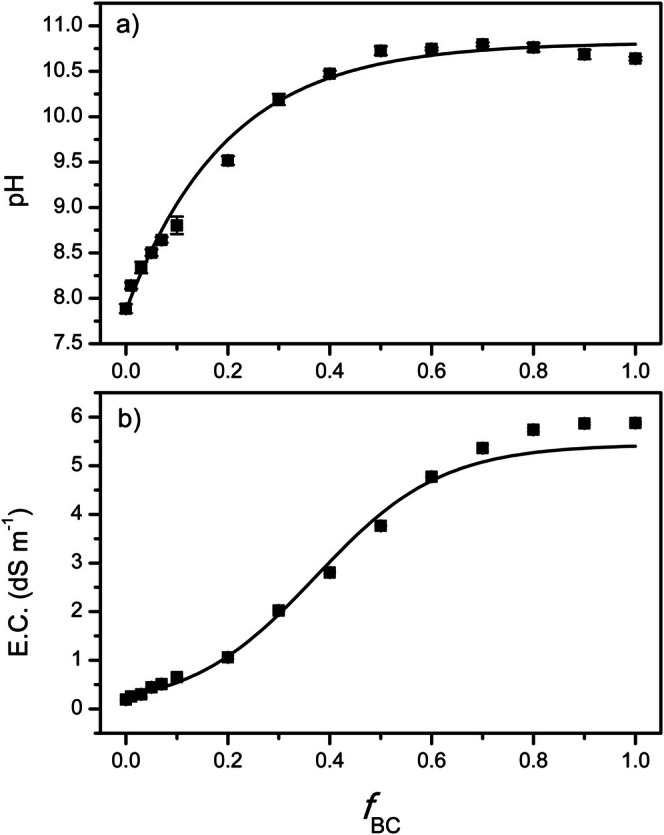
Variation of pH in soil–biochar mixtures as a function of biochar fraction (a) and electrical conductivity (EC) of soil–biochar mixtures as a function of biochar fraction (b).

The soil pH rises sharply upon biochar addition, increasing from ~7.9 to ~10.7 up to a biochar mass fraction (*f*_BC_) of about 0.5 (i.e., 50%). Beyond this threshold, further changes are negligible, producing a plateau that coincides with the pH of neat biochar (Figure 1a). The response is clearly nonlinear: small amounts of biochar lead to larger pH increments, whereas dosages above 50% yield progressively smaller variations until full saturation is reached. In other words, beyond this point the pH no longer changes, stabilizing around the characteristic value of the biochar itself. Figure 1a illustrates how the two pure materials define the extremes of the distribution: the unamended soil exhibits a sub‐alkaline pH (~7.9), while neat biochar is strongly alkaline (pH ~10.6). Mixtures with intermediate *f*_BC_ values show a gradual convergence toward the higher pH. The response is well captured by an exponential approach‐to‐plateau (Figure 1a).

The EC of soil–biochar mixtures also rises with increasing biochar fraction (Figure 1b). Unamended soil shows an initial EC of 0.2 dS m^−1^, whereas neat biochar exhibits a much higher conductivity (~5.8 dS m^−1^), attributable to its ash and soluble‐salt content. Progressive incorporation of biochar into the soil produces a neat monotone EC increase, again larger at low application rates. Once the biochar mass fraction (*f*_BC_) approaches ~0.8, the curve reaches a plateau at the value of pure biochar. Accordingly, the conductivity of intermediate mixtures is governed by the relative proportions of the two materials, rising nonlinearly from ~0.2 to ~5–6 dS m^−1^ as *f*_BC_ increases. Beyond this threshold, additional biochar does not produce significant EC gains, suggesting that the soil system becomes saturated in its capacity to adsorb or solubilize the ions released from the biochar. The dose–response follows a sigmoidal logistic trend with a finite plateau (Figure 1b).

### 3.2 Bulk Density

**FIGURE 2:**
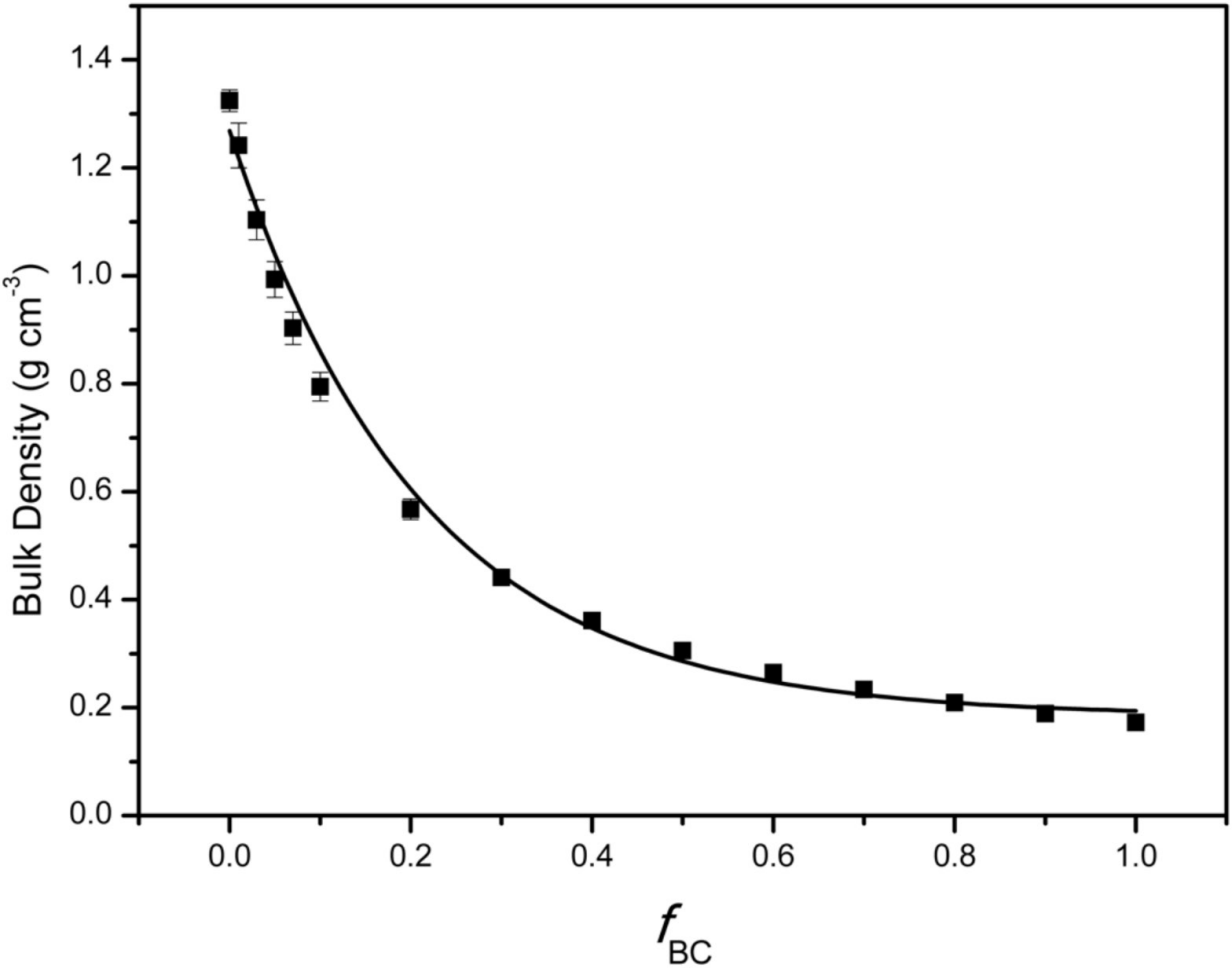
Changes in bulk density of the mixtures as a function of biochar addition.

The amendment with biochar caused a progressive reduction in the bulk density of the soil–biochar mixtures (Figure 2). The untreated soil displayed an initial value of 1.325 g cm^−3^—typical of a fine‐textured soil (Table 2)—whereas neat biochar had a markedly lower density (0.173 g cm^−3^) owing to its highly porous structure and low volumetric mass. Incorporating biochar systematically lowered the bulk density, with the steepest decline observed at the lower biochar mass fractions (*f*_BC_). This effect diminished at higher dosages, with a trend asymptotically approaching the density of pure biochar. For instance, at *f*_BC_ = 0.5 the bulk density was already substantially reduced; beyond this threshold, further decreases were more modest, converging toward values of only a few tenths of g cm^−3^.

All measured values are summarized in Table 2, which displays a gradual yet unmistakable decline. This behavior mirrors the markedly lower density of biochar relative to the mineral soil and indicates a proportional increase in the system's total porosity as the biochar content rises.

### 3.3 Structural and Surface Properties

**FIGURE 3:**
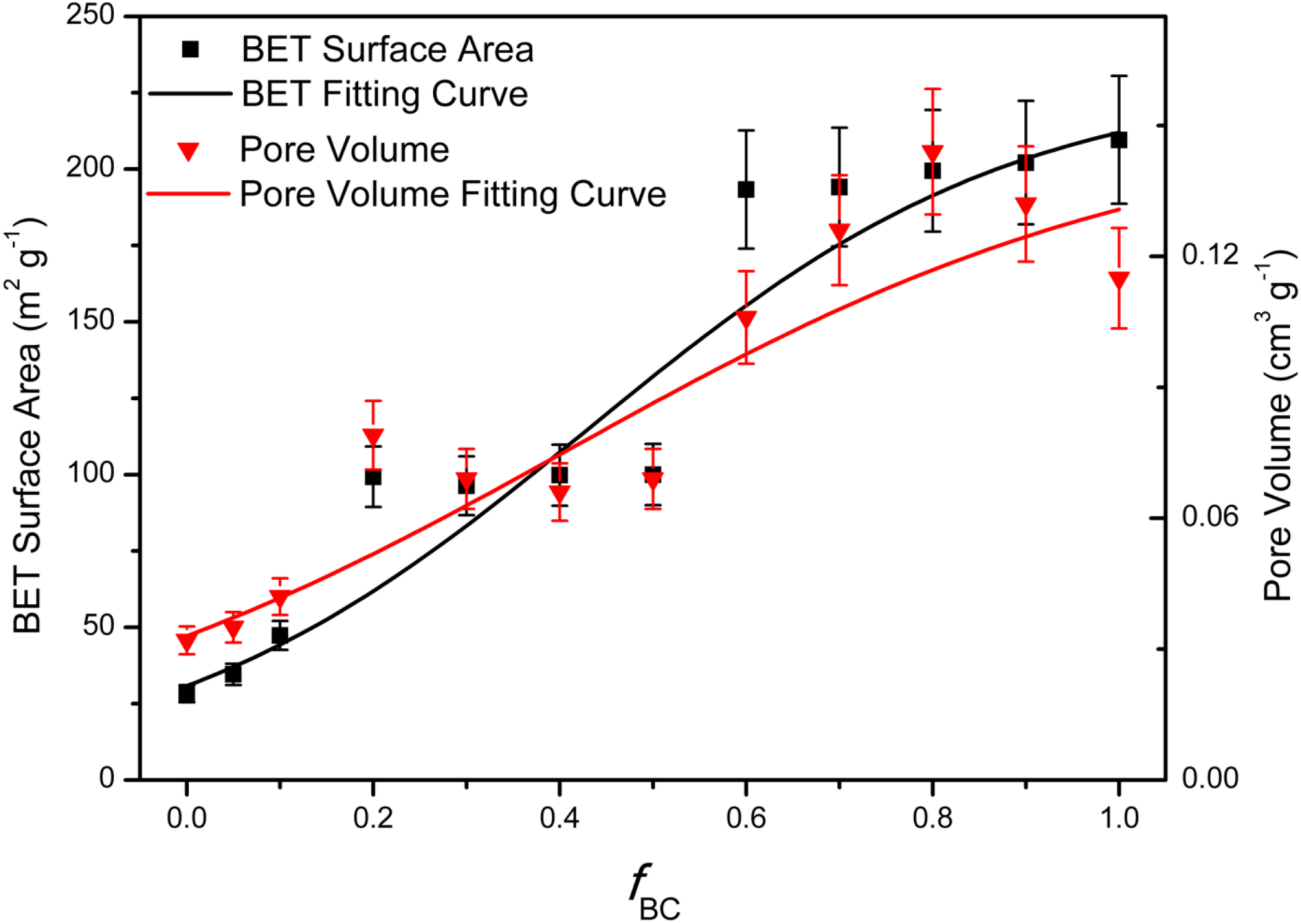
Increase in BET surface area (black) and total pore volume (red) of the mixtures with increasing *f*_BC_.

The structural properties of the amended soil—assessed in terms of specific surface area (BET) and total pore volume—showed marked increases as the biochar fraction rose (Figure 3). The specific surface area of the untreated soil was as large as 28.23 m^2^ g^−1^, whereas neat biochar reached 209.5 m^2^ g^−1^, according to its intrinsically porous architecture. Progressive incorporation of biochar into the soil led to a steady rise in the composite specific surface area, which gradually approached the characteristic value of the biochar. The trend was again not linear: a rapid increase occurred at low doses (*f*_BC_ < 0.1), followed by a first plateau between *f*_BC_ = 0.2 and 0.5. Beyond this threshold, the specific surface area increased further up to about *f*_BC_ = 0.6, where it stabilized at a second plateau.

This pattern indicates the presence of two discrete steps in the increase of specific surface area, presumably linked to successive phases of pore‐network expansion. A comparable trend was observed for the total pore volume with *f*_BC_ following a trajectory similar to that of the BET surface area. In the initial phase, biochar addition produced a marked rise in soil porosity; between *f*_BC_ = 0.2 and 0.5 a first level of structural saturation was reached, followed by a further—but modest—increment up to roughly *f*_BC_ ≈ 0.6. Beyond this threshold, the pore volume likewise tended to stabilize, converging toward the characteristic values of neat biochar.

These findings indicate that incorporating biochar markedly alters soil structure, increasing both the available specific surface area and the pore space (see Table 3), although the effect tends to saturate at high biochar fractions.

**TABLE 3:** BET surface area, total pore volume, and average pore radius of soil–biochar mixtures calculated from N_2_ adsorption isotherms.

$f_{\mathrm{BC}}$	BET surface area (m^2^ g^−1^)	Pore volume (cm^3^ g^−1^)	Pore radius (Å)
0	28.236	0.032	19.2
0.05	34.593	0.035	19.2
0.1	47.336	0.042	19.2
0.2	99.266	0.079	19.2
0.3	96.311	0.069	19.2
0.4	99.801	0.066	19.1
0.5	100.002	0.069	19.2
0.6	193.321	0.106	19.1
0.7	194.121	0.126	19.2
0.8	199.448	0.144	19.1
0.9	202.106	0.132	19.2
1	209.566	0.115	19.1

### 3.4 Water Holding Capacity (WHC)

**FIGURE 4:**
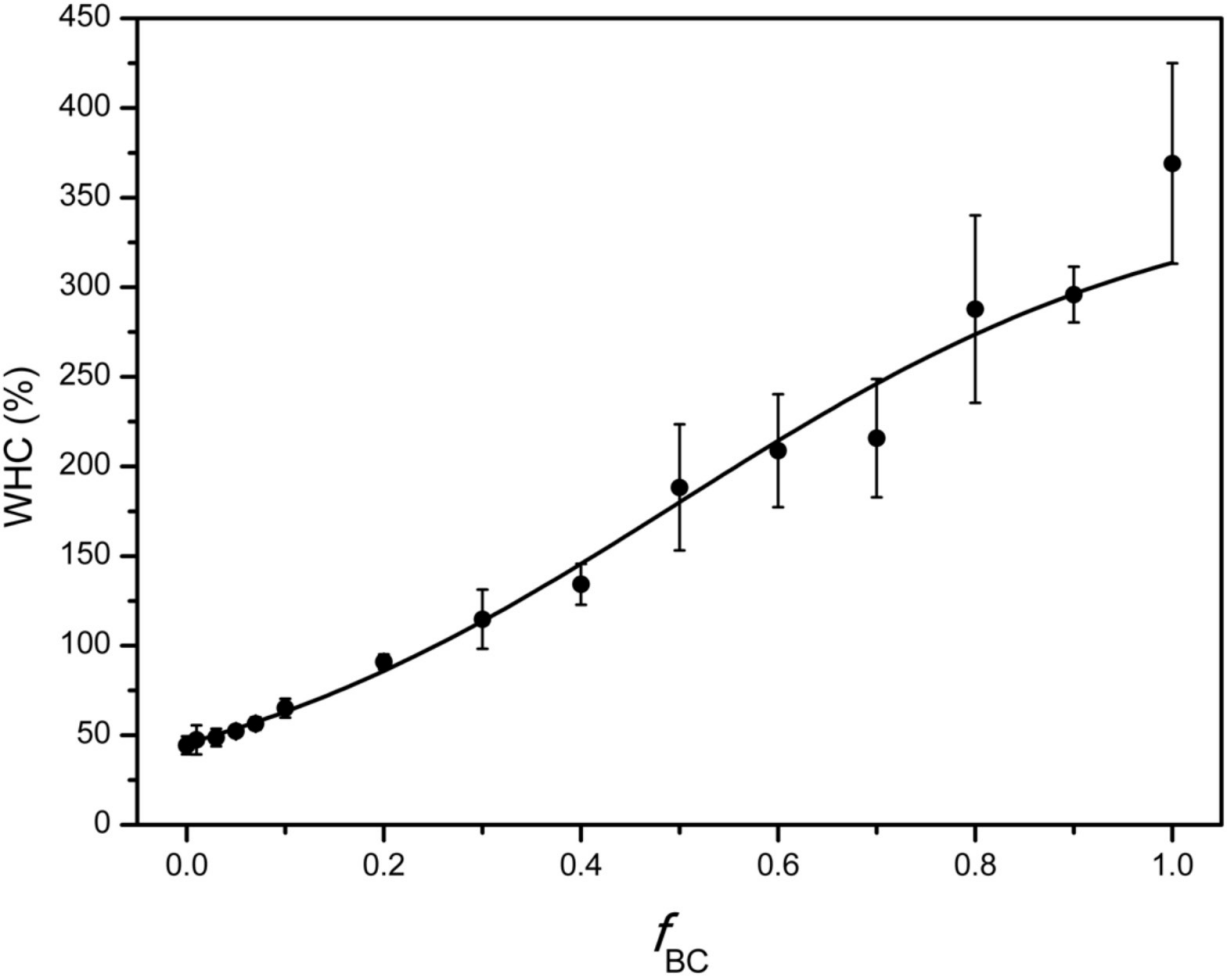
Water holding capacity (WHC) of soil–biochar mixtures as a function of *f*_BC_.

The addition of biochar markedly increased the soil's WHC. Unamended soil retained approximately 44% of its oven‐dry mass as water at field capacity (i.e., after saturation and free drainage), whereas neat biochar could hold about 310% of its own weight, underscoring the material's extraordinary ability to absorb and store water within its porous matrix (Figure 4).

Even small additions of biochar produced clear increases in the WHC of the soil–biochar mixtures. WHC increased monotonically with a saturating logistic trend (slogistic1), with an inflection at x_c_ = 0.50 ± 0.04 approaching a high‐dose plateau.

All measured values for the various mixtures are listed in Table 4.

**TABLE 4:** Water holding capacity (WHC, %) measured over four wetting–drying cycles for each mixture.

Fraction of biochar ( $f_{\mathrm{BC}}$)	WHC 1 (%)	WHC 2 (%)	WHC 3 (%)	WHC 4 (%)
0	44 ± 5	51 ± 4	48 ± 5	45 ± 5
0.01	48 ± 8	55 ± 3	52 ± 3	50 ± 3
0.03	49 ± 5	52 ± 5	51 ± 3	49 ± 3
0.05	52 ± 3	52 ± 2	51 ± 1	49 ± 2
0.07	56 ± 4	55.1 ± 0.5	53.8 ± 0.7	53.3 ± 0.5
0.1	65 ± 5	64 ± 1	59 ± 1	59 ± 2
0.2	91 ± 4	86 ± 4	80 ± 2	76 ± 5
0.3	115 ± 16	110 ± 27	103 ± 28	99 ± 24
0.4	134 ± 11	132 ± 18	122 ± 20	116 ± 15
0.5	188 ± 35	215 ± 7	198 ± 15	196 ± 8
0.6	209 ± 32	200 ± 27	176 ± 26	183 ± 24
0.7	216 ± 33	200 ± 19	189 ± 17	180 ± 12
0.8	288 ± 52	293 ± 31	283 ± 29	273 ± 39
0.9	296 ± 16	279 ± 5	283 ± 16	273 ± 28
1	309 ± 56	305 ± 18	312 ± 13	300 ± 17

### 3.5 Water Activity

**FIGURE 5:**
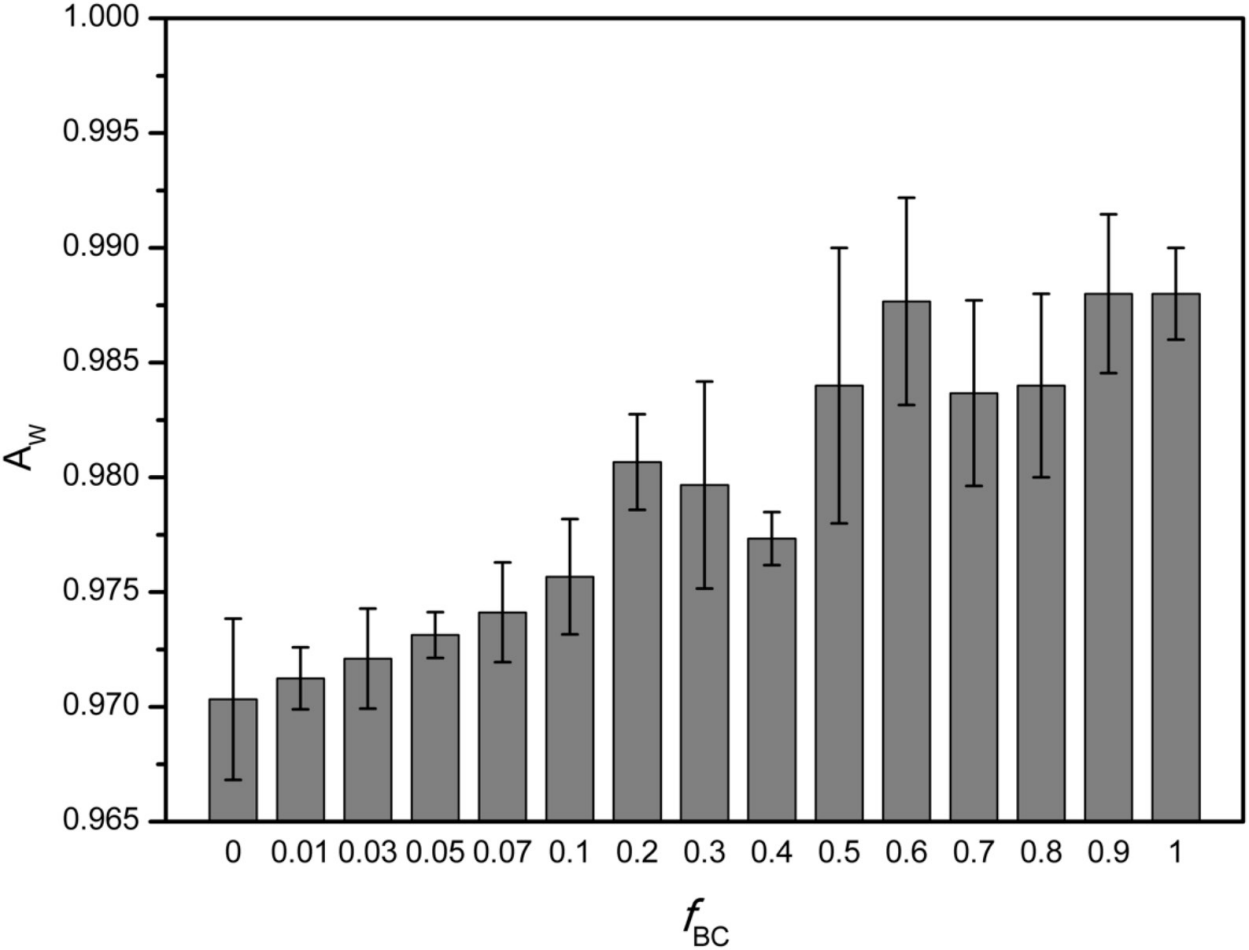
Water activity (A_w_) measured at field capacity.

The soil water activity ( $A_{W}$), measured at the moisture content corresponding to field capacity, displayed a modest rise with increasing biochar mass fraction (Figure 5).

In the unamended soil, $A_{W}$ was 0.970 (dimensionless, 0–1 scale), indicating that the pore water at field capacity was almost entirely available (for reference, saturated soil has $A_{W}$ = 1.0). Incorporating biochar into the mixtures raised $A_{W}$, signifying greater thermodynamic “freedom” of the water (i.e., a higher water chemical potential) within the system. The rise was most pronounced at low biochar fractions: for example, increasing *f*_BC_ from 0 to 0.10 produced an almost linear gain in $A_{W}$. Beyond this point, the trend became less regular, with some fluctuations up to *f*_BC_ ≈ 0.6, after which further additions led to stabilization. The sample composed of 100% biochar exhibited an $A_{W}$ of 0.988, approaching the theoretical limit.

### 3.6 Infrared Spectroscopy (FT‐IR)

**FIGURE 6:**
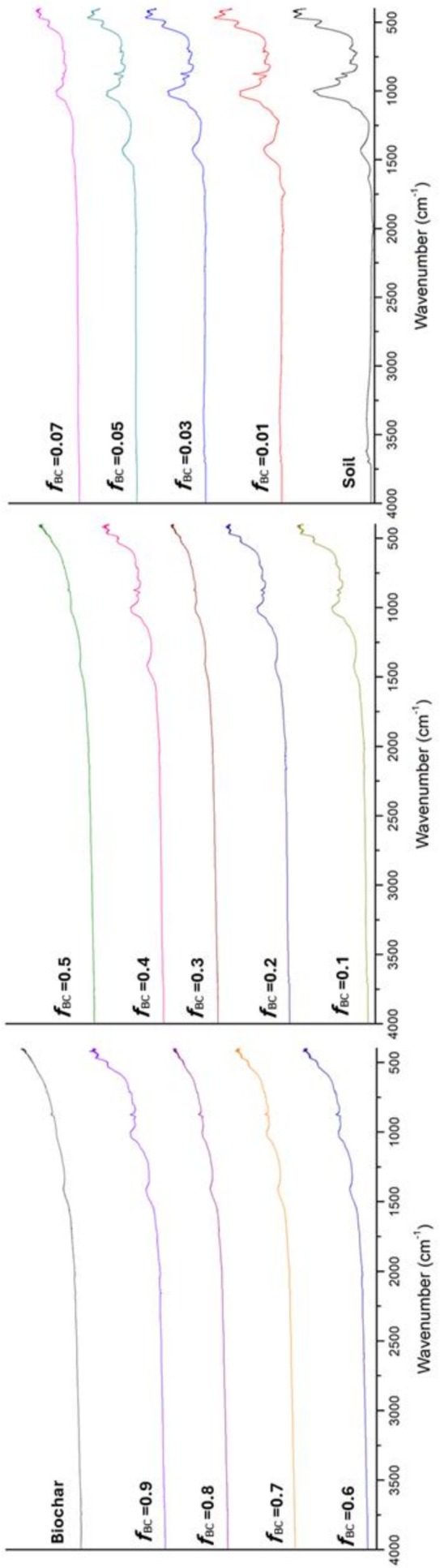
FT‐IR spectra of mixtures with increasing *f*_BC_.

FT‐IR analysis revealed marked changes in the functional composition of the mixtures as the biochar fraction (*f*_BC_) increased (Figure 6). In addition to the progressive decrease of the broad O–H stretching (~3400 cm^−1^) attributable to hydroxyl groups and adsorbed water [56], we observed the attenuation—and, above *f*_BC_ ≈ 0.07, the near disappearance—of the narrow inorganic O–H bands in the 3600–3700 cm^−1^ region, diagnostic of structural hydroxyls in phyllosilicates (e.g., kaolinite/halloysite at ~3695–3700 and ~3669–3652 cm^−1^; illite/smectite around ~3620–3630 cm^−1^) [57]. In the same *f*_BC_ range, the carbonate ν₃ band at ~1426 cm^−1^ (calcite) also weakens sharply. Concomitantly, bands characteristic of graphene‐like domains (~1580 cm^−1^, C=C; [51]) appear and intensify, pointing to the introduction of aromatic/graphitic functionalities inherent to biochar.

### 3.7 FFC‐NMR (Fast Field‐Cycling NMR)

**FIGURE 7:**
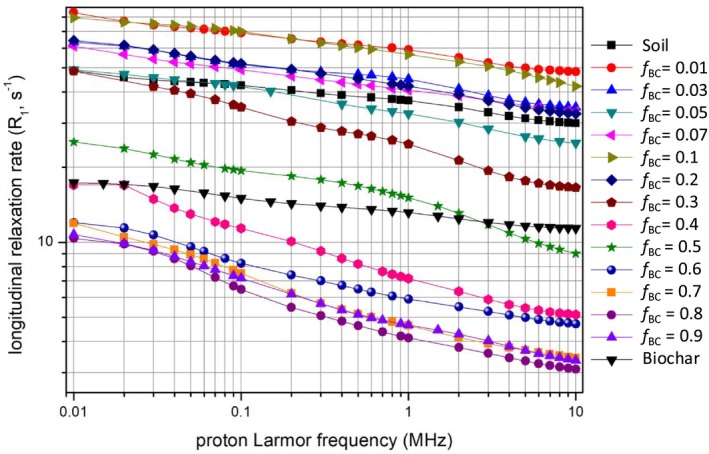
NMR dispersion (NMRD) profiles of water longitudinal relaxation rates (*R*_1_).

Fast Field‐Cycling NMR relaxometry (NMRD—Nuclear Magnetic Resonance Dispersion) offers a complementary view of how biochar amendment influences soil‐water dynamics (Figure 7).

The data reveal a clear yet nonlinear dose‐dependent trend: at low biochar fractions (*f*_BC_ ≤ 0.2) the longitudinal relaxation rate profile *R*_1_ of water protons remains similar to, or only slightly higher than, that of pristine soil. From *f*_BC_ = 0.3 onward, however, *R*_1_ declines sharply with increasing *f*_BC_, corresponding to a marked lengthening of the *T*_1_ times. Practically, once a certain biochar threshold is exceeded, water protons relax more slowly; at high biochar contents the water in the samples exhibits significantly longer *T*_1_ values than the unamended soil (i.e., lower *R*_1_). This behavior indicates that, at elevated biochar levels, fast surface‐controlled relaxation pathways contribute less to the overall relaxation—i.e., a larger fraction of the pore water is no longer in rapid exchange with strongly relaxing sites.

This dose‐dependent trend reveals a clear reversal beyond the threshold of *f*_BC_ ≈ 0.3. One might initially expect that adding a highly porous, hydrophilic material such as biochar would simply increase the sample's water content, eventually yielding “free” water and longer *T*_1_ values. Up to small biochar dosages, however, no significant *T*_1_ variations are detected—in fact, in some cases the *T*_1_ values are slightly shorter than those of soil without biochar. Once a certain biochar concentration is surpassed, the opposite effect becomes evident: high biochar dosages produce a pronounced lengthening of *T*_1_. For instance, soil amended to *f*_BC_ = 0.5 exhibits much longer *T*_1_ relaxation times than the untreated soil, approaching the behavior of the pure‐biochar sample at field capacity (which shows relaxation values intermediate among all samples).

The observed decrease in *R*_1_ (i.e., the increase in *T*_1_) with rising *f*_BC_ clearly indicates that biochar, beyond a certain amount, substantially alters the molecular environment of water in the soil. This effect is reproducible and was consistently observed across all specimens examined, confirming a robust biochar‐induced modification of soil‐water relaxation behavior.

**FIGURE 8:**
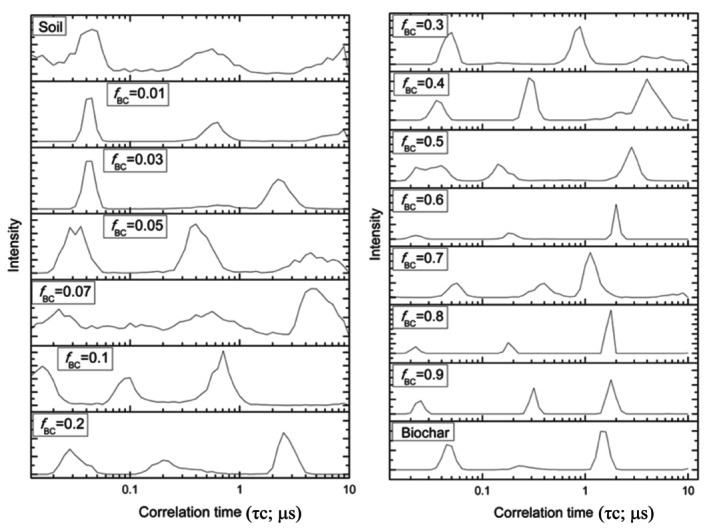
Distributions of water correlation times (*τ*_c_) obtained by Model‐Free analysis.

To quantify these findings, the NMRD profiles were processed according to a recently proposed heuristic free‐model analysis, yielding correlation‐time ( $\tau_{c}$) distributions for each sample (Figure 8); the corresponding numerical values are reported in Table 5. Correlation times *τ*_c_ characterise the timescale of molecular fluctuations (rotational reorientation and translational motion within pores): longer *τ*_c_ values reflect slower or more constrained dynamics, whereas shorter *τ*_c_ indicate more mobile water. In the following, τc distributions are used as the main descriptor of water mobility, while *T*_1_ is interpreted primarily as an integral measure of the overall efficiency of the relaxation pathways. Because *T*_1_ depends not only on pore geometry and surface‐to‐volume ratio but also on the abundance and speciation of paramagnetic centers, any relationship between *T*_1_ and pore size should be regarded as qualitative.

**TABLE 5:** Molecular correlation times (*τ*_c_) (μs) of water obtained from Model‐Free analysis of NMRD profiles for the various mixtures.

*f*_BC_	$\tau_{c1}\left(\mathrm{\mu s}\right)$	$\tau_{c2}\left(\mathrm{\mu s}\right)$	$\tau_{c3}\;\left(\mathrm{\mu s}\right)$
0	0.014	0.050	0.560
0.01	0.044	0.630	
0.03	0.045	0.700	2.230
0.05	0.035	0.400	4.470
0.07	0.022	0.560	4.460
0.1	0.015	0.100	0.700
0.2	0.028	0.250	2.250
0.3	0.050	0.900	4.470
0.4	0.035	0.280	3.980
0.5	0.031	0.140	2.820
0.6	0.025	0.200	2.820
0.7	0.056	0.350	1.120
0.8	0.022	0.177	1.770
0.9	0.025	0.310	1.770
1	0.050	0.280	1.580

ModelFree analysis shows that biochar amendment markedly affects these parameters. As *f*_BC_ increases, the $\tau_{c}$ distribution shifts toward higher values for a fraction of the water population: in samples with *f*_BC_ ≥ 0.3, components with significantly longer $\tau_{c}$ appear relative to the untreated soil. For example, in pure soil the dominant $\tau_{c}$ values all lie below 1 ms, whereas in high‐biochar soils $\tau_{c}$ values on the order of several milliseconds are detected. Simultaneously, the relative weight of the short‐ $\tau_{c}$ components decreases in the biochar mixtures. Thus, a subset of water molecules in biochar‐rich samples undergoes slower fluctuations.

The emergence of longer *τ*_c_ components indicates slower characteristic fluctuations for a fraction of the water population, i.e., the coexistence of distinct dynamical domains in biochar‐rich mixtures. At the same time, the overall lengthening of *T*_1_ (decrease of *R*_1_) indicates a reduced efficiency of surface‐controlled (inner‐sphere) relaxation, consistent with relaxation pathways becoming increasingly diffusion‐mediated (outer‐sphere). Thus, *τ*_c_ and *T*_1_ capture complementary aspects: *τ*_c_ reflects the timescale of molecular fluctuations, whereas *T*_1_ integrates the net efficiency of the available relaxation channels.

## 4 Discussion

### 4.1 Soil pH and EC: Buffering Effects and Optimal Application Ranges

The rise in soil pH observed after biochar addition aligns with the well‐documented liming effect of this amendment. Because biochar is typically alkaline owing to its ash content, it can neutralize soil acidity. The data show that small dosages already raise pH appreciably, which is advantageous for correcting acidic soils. However, beyond a certain dose the curve approaches a plateau, and further additions do not appreciably increase pH. In our system this plateau is reached around *f*_BC_ ≈ 0.5, which, if uniformly incorporated into the tilled topsoil, would correspond to an application rate of roughly 650 t ha^−1^—an agronomically unrealistic rate that we considered as an upper bound to characterise the full dose–response range. In practice, pH adjustment can be achieved well before this plateau: incorporation rates on the order of a few tens of tonnes per hectare (*f*_BC_ ≈ 0.1–0.2) already move the soil into the desirable pH range, while higher doses provide only diminishing additional benefits.

EC exhibits a similar dose‐dependent behavior with a clear threshold. Biochar introduces soluble salts and ash—indeed, the EC of neat biochar is roughly 30 × that of the parent soil—so amendment naturally increases the soil solution's ionic strength, especially at high application rates. At low‐to‐moderate rates this rise can be beneficial, reflecting an enrichment of the soil solution with nutrients such as K^+^, Ca^2+^, and carbonates released from the biochar. In the intermediate mixtures examined here, EC remained within a moderate range, indicating nutrient release without reaching problematic salinity levels for the specific combination of a high‐temperature wood‐derived biochar and a clay agricultural soil. This “moderate” EC increase is, however, specific to the materials used in this study. Biochars differ widely in ash content and solubility—for example, low‐pyrolysis‐temperature biochars generally contain more soluble salts and can cause much larger EC increases, as can biochars produced from manure or sludge feedstocks [58, 59]. Soil properties (texture, organic‐matter content, etc.) further modulate the outcome, so that a similar biochar dose can have very different effects in a sandy soil compared with a clayey one. When the biochar fraction becomes large enough, however, EC approaches ~5–6 dS m^−1^—a level at which a field soil would be classified as saline. Such salinity can impair seed germination and harm salt‐sensitive species. In this study, beyond *f*_BC_ ≈ 0.8 the EC curve flattens, implying that the soil cannot incorporate additional ions; excess salts likely remain undissolved, or the extra conductivity is offset by factors such as cation‐exchange saturation. Driving the biochar dose beyond this threshold therefore fails to increase the pool of soluble nutrients and instead risks osmotic stress or ion toxicity.

Previous studies have likewise reported initial EC increases proportional to biochar dose and ash content, followed by negative effects at excessive rates due to salt accumulation. The present results underscore the importance of dose optimization: a modest biochar input can moderately enhance EC—and thus nutrient availability—whereas a massive application can make the soil solution overly conductive. Consistent with this, from a management perspective application rates on the order of a few tens of tonnes per hectare (≈ 20–50 t ha^−1^, corresponding to *f*_BC_ ≈ 0.1–0.2) emerge as a reasonable target range: they enhance pH and EC while remaining safely below the salinity and alkalinity plateaus observed at much higher, purely experimental doses.

### 4.2 Physical Soil Properties: Bulk Density, Porosity, and Structure

One of the clearest benefits emerging from this study is the improvement in soil physical properties—most notably the reduction in bulk density and the concomitant increase in total porosity. Incorporating a lightweight, highly porous material such as biochar into a dense clay soil resulted in an overall structure that is markedly softer and better aerated. This outcome agrees with the literature: numerous studies have reported density decreases and porosity gains in biochar‐amended soils, attributing them to both a dilution effect (lower mass per unit volume due to the biochar) and the development of more stable, macropore‐rich aggregates [9, 30].

In the experiments presented here, bulk density dropped sharply even after modest additions of biochar and progressively approached the intrinsic density of the biochar at the highest dosages. Consequently, the total soil porosity increased: the biochar introduced a significant additional porosity, particularly within the mesopore and macropore ranges, as indicated by BET/BJH analyses (Table 3). Both greater porosity and lower density imply a soil that is easier to till and better aerated. Practically, this translates into reduced compaction and lower mechanical resistance to root penetration.

The biochar effect on physical properties is, however, dose‐dependent and subject to diminishing returns. The data show pronounced structural improvements (lower density, higher porosity) up to a threshold beyond which marginal gains taper off and potential drawbacks may arise (e.g., excessive friability or loss of cohesion). From a management perspective, relatively low‐to‐moderate application rates (≈ 1–5 wt %, depending on soil type) are likely sufficient to secure most structural benefits—improved workability, greater macroporosity, enhanced infiltration—without compromising aggregate stability. Such rates correspond to a few tens of tonnes per hectare, levels already tested successfully in agronomic trials. Much higher dosages, by contrast, make the mixture behave increasingly like pure biochar: an extremely light and porous material that lacks the cohesion and bearing capacity characteristic of a mineral soil. An excess of biochar could therefore, in theory, yield a soil that is overly soft, prone to erosion, and incapable of providing structural support for plants. In short, an optimal window again emerges: small additions markedly improve soil structure, whereas extreme additions are neither practical nor desirable.

### 4.3 Water Retention and Availability: Benefits and Potential Drawbacks

The improvements in WHC observed after biochar addition represent one of the most promising practical advantages of using biochar as an amendment. The almost linear rise in WHC with *f*_BC_ demonstrates that biochar can act as a potent soil water reservoir, absorbing moisture and retaining it against gravitational drainage. This is particularly valuable in dry climates where soils suffer from poor water retention. In our study, neat biochar held more than 300% of its own dry mass in water (vs. ~44% for the unamended soil); even small percentages of biochar mixed into the soil significantly increased the water retained at field capacity. Numerous reports have documented similar increases, especially in sandy or degraded soils with low initial field capacity [60, 61, 62]. Here—despite working with a clay soil that already exhibits good retention—a marked rise was still detected, showing that the biochar effect is not limited to coarse textures.

Unlike pH and EC, a clear saturation point for WHC was not identified within the dosage range examined: more biochar simply meant more stored water. From a purely hydrological viewpoint, then, biochar continues to provide benefits virtually up to the level of pure biochar. Yet it is also essential to consider how this extra water is held and how much is actually available to plants.

Measurements of water activity ( $A_{W}$) offer a first clue: a slight increase was recorded with biochar addition. This indicates that the extra water is held less tightly—closer to “free” water—than in the pure soil. In theory, a soil containing more water and having a higher $A_{W}$ should supply crops with a larger, more accessible moisture reserve, delaying wilting under drought. This scenario is confirmed only up to a point by the present data: higher WHC and a modestly higher $A_{W}$ together portray a soil that not only retains more water but also makes it somewhat more available.

Two important considerations, however, must be kept in mind. First, the observed rise in $A_{W}$ is relatively small (from 0.970 to 0.988): in both cases the water is still held under tension (field capacity, not free water). Although biochar adds a large volume of water, part of it is stored in very fine pores. If a significant fraction of this moisture is trapped in micropores inside the biochar particles, it may not be easily extractable by plant roots, becoming an effectively “unavailable” reservoir. Indeed, some literature indicates that, while biochar raises water content at field capacity, the increment in plant‐available water (i.e., the fraction between field capacity and permanent wilting point) is not always proportional—sometimes negligible—when the added water is confined to pores too small to be exploited by roots. In the present work, the wilting point was not measured directly, but FFC‐NMR data (see later) indicate that, in the amended mixtures, a larger fraction of the pore water relaxes through weaker surface‐controlled pathways (i.e., with a reduced contribution from strongly relaxing sites). This qualitative evidence suggests that at least part of the additional water retained at field capacity is more weakly bound and may therefore remain accessible to plant uptake, rather than being entirely confined within strongly sorbing micropores.

As a second consideration, it should be remarked that retaining too much water can negatively affect soil aeration under saturated conditions. A soil that stores almost all possible water ( $A_{W}$ near 1) risks remaining saturated for long periods after rainfall, reducing the air‐filled porosity essential for roots and microorganisms. In our experiment, at the highest biochar levels $A_{W}$ approached ~0.99, indicating that the soil was almost saturated at field capacity. In practice, after heavy rain a biochar‐rich soil might fail to absorb further water, resulting in immediate runoff. Beyond a certain moisture threshold, every additional precipitation event translates into surface flow because no pore space remains to store it. This heightened runoff risk (despite the initially good infiltration promoted by biochar) is a potential side‐effect. Moreover, a soil that stays saturated creates anaerobic conditions harmful to roots and biota: $A_{W}$ values close to 0.99 signal very wet soil, and if such moisture persists hypoxic stress or root rot can develop.

Fortunately, biochar generally improves drainage as well, thanks to increased macroporosity, partially offsetting the saturation risk. In this study, despite the very high water retention at elevated *f*_BC_, the improved structure also enhanced infiltration and drainage. Some reports note that clay soils can suffer reduced infiltration if amended with fine particles (which may clog pores) [63, 64]; however, the mixed particle size of the biochar and its high internal porosity appear to have avoided this problem. Indeed, both infiltration (via greater macroporosity and aggregate structure) and field capacity (via biochar microporosity) increased simultaneously—highlighting a distinctive feature of biochar: it can improve both ends of the soil water balance, storing more water while still permitting efficient percolation of excess moisture.

In short, for soil‐water management, the optimal biochar rate will be the one that maximizes field capacity and plant‐available water without leaving the soil chronically too wet or poorly aerated. Many studies suggest that moderate applications (≈ 2–5 wt %) are sufficient to sharply improve water retention in sandy or silty soils, aiding crops during dry spells, while higher doses yield diminishing returns [65, 66, 67]. Even in the clay soil examined here—already characterized by good retention—biochar conferred incremental benefits, but beyond ~10 wt % clear signs of diminishing gains appeared (e.g., $A_{W}$ plateau and greater data variability). In practice, a reasonable recommendation would be not to exceed ~10 wt % (≈100 t ha^−1^ in the top 5 cm) if the goal is to enhance water storage without incurring saturation problems. Naturally, the optimum varies with texture: sandy soils may tolerate and even require more biochar to reach saturation, whereas fine‐textured soils attain their hydrological ceiling sooner.

### 4.4 Molecular Insights From FT‐IR and FFC‐NMR

FT‐IR spectroscopy was used to track how the surface chemistry of the soil–biochar mixtures evolves with increasing *f*_BC_ and to relate these changes to the water‐dynamics patterns revealed by FFC‐NMR. As biochar is added, the spectra show a progressive replacement of bands associated with hydrophilic mineral and organic components by pyrogenic carbon functionalities. In particular, the broad O–H stretching band (~3400 cm^−1^, hydroxyls and adsorbed water) decreases in intensity, while bands characteristic of graphene‐like domains (e.g., C=C at ~1580 cm^−1^) [68] and oxygenated pyrogenic groups become increasingly prominent. From inspection of the spectra, the response is clearly nonlinear, with a threshold‐like behavior: already at *f*_BC_ ≈ 0.07 the narrow structural O–H bands in the 3600–3700 cm^−1^ region (phyllosilicate hydroxyls) [69] and the calcite *ν*_3_ band at ~1426 cm^−1^ [70] are strongly attenuated, and most sharp peaks in the 1000–1030 cm^−1^ Si–O–Si stretching region [71] and in the fingerprint zone (including bands assigned to native organic matter at ~790 cm^−1^) [72] become barely detectable. This widespread attenuation is best explained by the strong ability of graphene‐like, Csp^2^‐rich domains in the biochar to absorb IR radiation, effectively reducing the penetration depth of the beam into the mineral phase and producing a spectral dilution/masking of mineral and organic signals rather than a loss of the underlying phases. In other words, the mineral –OH groups, carbonates and silicate frameworks are still present in the mixtures, but their contribution to the ATR‐FT‐IR spectrum is progressively overshadowed by the strongly absorbing carbonaceous matrix as *f*_BC_ increases. Beyond the threshold, the spectra are therefore dominated by aromatic/graphitic features and a subset of oxidised pyrogenic groups, consistent with a surface chemistry in which water interacts predominantly with biochar‐derived pores and π‐rich domains. This reorganisation of the functional fingerprint is in line with the observed increase in WHC and with the FFC‐NMR evidence for a larger fraction of water residing in biochar‐controlled pore domains where relaxation is less dominated by efficient surface‐controlled pathways. FFC‐NMR relaxometry offers a complementary picture focused on water dynamics. As the biochar dose increases, longitudinal relaxation times (*T*_1_) become markedly longer, indicating that water in the amended soil is, on average, less efficiently relaxed through surface‐controlled mechanisms. This change, evident beyond *f*_BC_ ≈ 0.3, can be interpreted in terms of two fundamental relaxation pathways in porous systems—i.e., (i) Outer‐sphere (diffusive) relaxation: water molecules diffuse freely through the pores and relax via weak dipole–dipole interactions without forming stable surface bonds, so that fluctuations in local magnetic fields near pore walls suffice to produce partial relaxation; and (ii) Inner‐sphere (surface‐adsorption) relaxation: water forms transient bonds at surface sites, where protons relax rapidly through strong interactions with functional groups or paramagnetic centers.

For biochar‐amended soils, the data point to a predominance of the outer‐sphere mechanism: rising *T*_1_ values imply that water interacts less frequently and less strongly with solid surfaces, despite being present in larger amounts. This behavior is consistent with biochar's porous architecture and the relative scarcity of magnetically active centers or functional groups able to drive efficient relaxation. Even when water adheres to biochar surfaces (inner‐sphere), relaxation remains slow, probably owing to the lower density of relaxing sites compared with mineral surfaces. Overall, the extra water held by biochar appears more weakly coupled to relaxation‐active surface sites, supporting the view that a substantial fraction of this water is not tightly immobilized and can effectively contribute to the macroscopically observed increase in water availability.

Agronomically, these findings suggest that biochar enhances soil water retention without tightly immobilizing the water on surfaces. The moisture stored in biochar pores is held primarily by capillarity, not by strong surface adsorption, making it more easily exchangeable with the surrounding soil solution and accessible to plants. Hence, biochar can raise the soil's water reserve while preserving the fluidity required for biological uptake.

This behavior is consistent with the nonlinear trend in water availability: low biochar doses have modest effects, whereas higher doses markedly increase stored water but with diminishing returns. The threshold‐like response likely reflects the progressive filling of less‐effective pores that retain excess moisture without necessarily benefiting plant physiology. In summary, FFC‐NMR indicates that high biochar rates reorganise pore structure and surface chemistry in a way that maximises water retention without sacrificing dynamic availability for soil organisms and roots.

## 5 Conclusion

This study mapped the dose–response of a clay agricultural soil to a poplar biochar by combining standard soil analyses, FT‐IR spectroscopy and FFC‐NMR relaxometry on fifteen mixtures spanning 0 ≤ *f*_BC_ ≤ 1. All measured properties showed nonlinear trends with thresholds and plateaus, rather than proportional changes with dose. Soil pH and EC rose rapidly at low *f*_BC_ and then approached the respective biochar values (≈ 10.7 and ≈ 5–6 dS m^−1^) as *f*_BC_ approached ≈ 0.5 and ≈ 0.8, respectively. Bulk density decreased strongly at low *f*_BC_, whereas specific surface area, pore volume and WHC increased with saturating behavior. FFC‐NMR indicated that above *f*_BC_ ≈ 0.3 an increasing fraction of pore water relaxes more slowly, consistent with storage in less strongly relaxing microenvironments.

From an agronomic standpoint, these dose–response functions indicate that, for this soil–biochar system, most of the useful changes are obtained at moderate biochar fractions. In the range *f*_BC_ ≈ 0.1–0.2 (≈ 10%–20% w/w, corresponding to ≈ 70–170 t ha^−1^ in the top 5 cm), pH is already shifted toward the biochar value but remains well below the alkaline plateau, EC is still far from its saturation level, bulk density is substantially reduced and WHC is clearly increased, while the mixture retains the mechanical behavior of a soil rather than that of pure biochar. Higher fractions mainly drive the system toward the limiting properties of the biochar itself, with only limited additional agronomic benefit under the conditions explored here.

Finally, our multi‐technique investigation indicates that biochar alters soil water dynamics along the dose gradient by progressively shifting the dominant relaxation behavior from surface‐controlled pathways toward pore‐storage mechanisms. The use of a stretched‐exponential model to describe intrinsically multiexponential *T*_1_ decays provided a robust effective metric to compare overall relaxation behavior across the biochar dose series. Subsequent ModelFree analysis of the NMRD profiles revealed the emergence of longer correlation‐time (*τ*_c_) components at higher biochar contents, consistent with an increased contribution of water residing within biochar‐controlled pore domains and with relaxation pathways increasingly governed by diffusion‐controlled (outer‐sphere) mechanisms. Although more complex deconvolution of the inverse Laplace transform (ILT) patterns could, in principle, resolve finer water pools, we did not pursue ILT‐based deconvolution because overlapping components would not allow an objective, model‐independent interpretation. Moreover, our approach captured the systematic, dose‐dependent transition in water dynamics. Together with FT‐IR and macroscopic results, these trends support the view that the enhanced water retention primarily reflects capillary storage within biochar's pore network rather than strong surface adsorption, thereby keeping a substantial fraction of retained water weakly surface‐coupled and potentially available, consistent with the slight increase in water activity (Aw).

## Supporting information


**Table S1:** MRC_70077‐sup‐0001‐Supporting_Information.docx. *T*_1_ values of water protons in soil–biochar mixtures at different proton Larmor frequencies (*ν*_1_) and biochar mass fractions (*f*_BC_), measured by FFC‐NMR relaxometry and used to construct the NMRD profiles *R*_1_(*ν*ₗ) = 1/*T*_1_ in the main text.
**Figure S1:** Pulse sequence to acquire a fast field cycling NMR relaxometry experiment. The reader is addressed to the main text for the details about the sequence.

## Data Availability

The data that support the findings of this study are available from the corresponding author upon reasonable request.
