# Supplementary material for: Dose‐Dependent Effects of Biochar on Soil Revealed by Fast Field‐Cycling (FFC) NMR: From Molecular Water Dynamics to Soil Functionality
Source: Magn Reson Chem. 2025 Dec 31;64(3):349–64. doi: 10.1002/mrc.70077 (PMC12867593; doi:10.1002/mrc.70077)
Supplement: Supplementary file 1 — Table S1: MRC_70077‐sup‐0001‐Supporting_Information.docx. T 1 values of water protons in soil–biochar mixtures at different proton Larmor frequencies (ν 1) and biochar mass fractions (f BC), measured by FFC‐NMR relaxometry and used to construct the NMRD profiles R 1(νₗ) = 1/T 1 in the main text. Figure S1: Pulse sequence to acquire a fast field cycling NMR relaxometry experiment. The reader is addressed to the main text for the details about the sequence. [file MRC-64-349-s001.docx]

# **Dose-dependent effects of biochar on soil revealed by Fast Field-Cycling (FFC) NMR: from molecular water dynamics to soil functionality.**

Calogero Librici^1*^, Paola Bambina^1^, Ettore Madonia^1^, Veronica Ciaramitano^2^, Delia Francesca Chillura Martino^2^, Paolo Lo Meo^2^, Pellegrino Conte^1^

1. Dipartimento di Scienze Agrarie, Alimentari e Forestali (SAAF), Università degli Studi di Palermo, v.le delle Scienze ed. 4, 90144 Palermo (Italy)
2. Dipartimento di Scienze e Tecnologie Biologiche Chimiche e Farmaceutiche (STeBiCeF), Università degli Studi di Palermo, v.le delle Scienze ed. 16, 90144 Palermo (Italy)

*. Corresponding author: calogero.librici@unipa.it

Methods S1. FFC-NMR details

Figure S1 reports the typical sequence used to perform an FFC NMR relaxometry experiment (Conte and Lo Meo, 2020). Briefly, the experiment involves the application of a polarization (**B_pol_**), a relaxation (**B_rlx_**), and an acquisition (**B_acq_**) field. The former can be either null or non-null. When **B_pol_** is null, a non-polarized (NP) sequence is obtained, while when it is non-null, a pre-polarized (PP) sequence is achieved. In the PP sequence, **B_pol_** (having, in this study, a proton Larmor frequency of 9 MHz) is applied for a duration referred to as polarization time (T_pol_) whose value must be ≥ $4T_{1}^{*}$, where $T_{1}^{*}$ is the expected longitudinal relaxation time for the sample under investigation (Conte, 2021). Pre-polarization is necessary to generate magnetization, which then evolves to reach a new equilibrium condition under the influence of the relaxation field (**B_rlx_**) applied for a variable period of time, denoted as $\tau$ (Figure S1). In particular, **B_rlx_** intensity is varied in the proton Larmor frequency range 0.01-10 MHz, while $\tau$ is changed in the interval $0.01T_{1}^{*}\leq\tau\leq4T_{1}^{*}$, having $T_{1}^{*}$ the same meaning as reported above. After the application of **B_rlx_**, the magnetic field is

switched to a new value (indicated as **B_acq_**, that, in the present study, corresponds to the proton Larmor frequency of 7.2 MHz), while a 90° pulse is applied to generate the observable magnetization, which allows free induction decay (FID) acquisition.

The use of the PP sequence is recommended when the intensity of the relaxation field is too low and sensitivity enhancement is needed. The crossover field between the PP and NP sequences is usually empirically determined at $\nu_{rlx}=\frac{\nu_{pol}}{2}$ (Anoardo et al., 2001), where $\nu_{rlx}$, and $\nu_{pol}$ are the proton Larmor frequencies of the relaxation and polarization fields, respectively. In the present study, the crossover field was fixed at the proton Larmor frequency of 3 MHz. In Figure S1, the switching time (SWT) is also indicated. This is the time required for the transition between different magnetic field intensity values. SWT is typically set at approximately 3 ms. Shorter SWT values can only be used if specific electronic precautions are implemented (Kimmich and Anoardo, 2004).

Following data acquisition, decay (via PP sequence), and recovery (via NP sequence) curves are achieved. Equations (1), for the PP experiment, and (2), for the NP experiments, are, then, applied to estimate the $T_{1}$ values at each **B_rlx_** (Conte and Lo Meo, 2020):

$M\left( \tau\right)=a+b\left\{ exp\left[ -\left( \frac{\tau}{T_{1}} \right)^{k} \right] \right\}$ (1)

$M\left( \tau\right)=a+b\left\{ 1-exp\left[ -\left( \frac{\tau}{T_{1}} \right)^{k} \right] \right\}$ (2)

here, $M\left( \tau\right)$ is the area of the FID (i.e., the intensity of the magnetization) at each selected $\tau$ value (Figure 1); $a$ is the offset; $b$ is the magnetization intensity modulated by the longitudinal relaxation time, $T_{1}$; $k$ a heterogeneity parameter related to the stretched shape of the decay/recovery curves.

The $T_{1}$ values obtained by equations (1), and (2) were used to calculate the longitudinal relaxation rates ($R_{1}=T_{1}^{-1}$), which were, in turn, applied to obtain the nuclear magnetic resonance dispersion (NMRD) curves (i.e., $R_{1}$-vs-$\nu_{rlx}$ graphs). The latter were fitted by applying the ModelFree software package (downloadable at <https://site.unibo.it/softwaredicam/en/modelfree>) which provided also the distribution of correlation times ($\tau_{c}$). The latter measure the time needed for a molecule to rotate 1 rad or to move along a distance equal to its gyration radius. Hence, the longer the $\tau_{c}$, the more immobilized is the molecular system. Conversely, as $\tau_{c}$ decreases, the molecular system becomes more mobile and less constrained. For the detailed theory about the ModelFree software package please refer to Lo Meo et al. (2021), Landi et al. (2023), and Bortolotti et al. (2024).

Table S1.

T₁ values of water protons in soil–biochar mixtures at different proton Larmor frequencies (νₗ) and biochar mass fractions (*f*_BC_), measured by FFC-NMR relaxometry and used to construct the NMRD profiles R₁(νₗ) = 1/T₁ in the main text.

| proton Larmor frequency (MHz) | longitudinal relaxation rate R_1_(s^-1^) | proton Larmor frequency (MHz) | longitudinal relaxation rate R_1_(s^-1^) | proton Larmor frequency (MHz) | longitudinal relaxation rate R_1_(s^-1^) | proton Larmor frequency (MHz) | longitudinal relaxation rate R_1_(s^-1^) | proton Larmor frequency (MHz) | longitudinal relaxation rate R_1_(s^-1^) |
| --- | --- | --- | --- | --- | --- | --- | --- | --- | --- |
| Soil | | 0.01 *f_BC_* | | 0.03 *f_BC_* | | 0.05 *f_BC_* | | 0.07 *f_BC_* | |
| 10.00 | 30 ± 1 | 10.00 | 49.4 ± 0.4 | 10.00 | 36.7 ± 0.6 | 10.00 | 25.6 ± 1.1 | 10.00 | 33.6 ± 0.4 |
| 9.00 | 29.8 ± 1.1 | 9.00 | 49.5 ± 0.7 | 9.00 | 35.6 ± 0.5 | 8.00 | 25.4 ± 0.5 | 9.00 | 33.7 ± 0.6 |
| 8.00 | 30.1 ± 1.1 | 8.00 | 49.4 ± 0.9 | 8.00 | 34.6 ± 0.7 | 6.00 | 24.9 ± 0.6 | 6.00 | 34.7 ± 0.7 |
| 7.00 | 30.2 ± 0.9 | 7.00 | 49.6 ± 1.1 | 7.00 | 34.6 ± 0.9 | 5.00 | 26.6 ± 0.6 | 3.00 | 37.3 ± 0.9 |
| 6.00 | 31.9 ± 1.0 | 6.00 | 48.0 ± 0.7 | 6.00 | 34.3 ± 0.9 | 3.00 | 28.6 ± 1.9 | 2.00 | 38 ± 1 |
| 5.00 | 31.5 ± 0.3 | 5.00 | 47.9 ± 1.5 | 5.00 | 36.2 ± 1.0 | 2.00 | 30.7 ± 1.1 | 1.00 | 41 ± 1 |
| 4.00 | 31.0 ± 6.5 | 4.00 | 48.7 ± 1.3 | 4.00 | 36.6 ± 1.5 | 1.00 | 32.6 ± 0.8 | 0.80 | 41.3 ± 0.9 |
| 3.00 | 32.7 ± 1.6 | 3.00 | 52.1 ± 1.5 | 3.00 | 39.4 ± 1.3 | 0.80 | 33.5 ± 1.1 | 0.60 | 42.3 ± 0.9 |
| 2.00 | 35.9 ± 1.4 | 2.00 | 57.7 ± 1.3 | 2.00 | 41.8 ± 1.3 | 0.60 | 34.3 ± 0.8 | 0.50 | 43.3 ± 0.9 |
| 1.00 | 36.7 ± 1.1 | 1.00 | 59.7 ± 1.1 | 1.00 | 44.8 ± 1.0 | 0.40 | 35.6 ± 0.7 | 0.40 | 44 ± 1 |
| 0.90 | 37.1 ± 1.5 | 0.80 | 59.7 ± 1.2 | 0.80 | 46.3 ± 1.0 | 0.15 | 40.4 ± 1.1 | 0.30 | 44.9 ± 0.9 |
| 0.80 | 37.2 ± 2.5 | 0.50 | 61.0 ± 1.3 | 0.60 | 46.8 ± 1.4 | 0.09 | 42.4 ± 1.1 | 0.20 | 46.3 ± 0.9 |
| 0.60 | 38.0 ± 1.4 | 0.40 | 62.3 ± 1.4 | 0.50 | 47.0 ± 1.1 | 0.08 | 43.1 ± 1.4 | 0.10 | 49.3 ± 1.2 |
| 0.40 | 39.1 ± 1.2 | 0.30 | 64.0 ± 1.4 | 0.30 | 48.3 ± 1.0 | 0.07 | 43.4 ± 1.3 | 0.07 | 50.5 ± 1.3 |
| 0.30 | 40 ± 1 | 0.20 | 65.6 ± 1.1 | 0.20 | 48.8 ± 1.1 | 0.04 | 44.8 ± 1.3 | 0.05 | 52.5 ± 1.2 |
| 0.20 | 39.9 ± 1.6 | 0.10 | 67.6 ± 1.4 | 0.10 | 51.6 ± 1.1 | 0.03 | 45.2 ± 1.1 | 0.04 | 52 ± 1 |
| 0.10 | 43.0 ± 1.7 | 0.08 | 72.0 ± 1.5 | 0.09 | 52.3 ± 1.3 | 0.02 | 47.6 ± 0.9 | 0.03 | 54.1 ± 1.1 |
| 0.08 | 43.1 ± 1.1 | 0.07 | 71.4 ± 1.8 | 0.07 | 54.2 ± 1.1 | 0.01 | 49.1 ± 1.5 | 0.02 | 57.2 ± 1.3 |
| 0.06 | 43.1 ± 0.9 | 0.06 | 71.2 ± 1.5 | 0.05 | 54.9 ± 1.4 |  |  | 0.01 | 61.1 ± 1.3 |
| 0.05 | 44.6 ± 1.2 | 0.05 | 70.7 ± 1.3 | 0.04 | 55.5 ± 1.2 |  |  |  |  |
| 0.04 | 43.2 ± 1.2 | 0.04 | 73.3 ± 1.3 | 0.03 | 59.1 ± 1.5 |  |  |  |  |
| 0.03 | 45.1 ± 1.1 | 0.03 | 75.1 ± 1.3 | 0.02 | 62.7 ± 1.8 |  |  |  |  |
| 0.02 | 45.9 ± 1.2 | 0.02 | 76.6 ± 1.4 | 0.01 | 62.6 ± 2.2 |  |  |  |  |
| 0.01 | 48.8 ± 1.3 | 0.01 | 83.5 ± 2.1 |  |  |  |  |  |  |
|  |  |  |  |  |  |  |  |  |  |
| proton Larmor frequency (MHz) | longitudinal relaxation rate R_1_(s^-1^) | proton Larmor frequency (MHz) | longitudinal relaxation rate R_1_(s^-1^)) | proton Larmor frequency (MHz) | longitudinal relaxation rate R_1_(s^-1^) | proton Larmor frequency (MHz) | longitudinal relaxation rate R_1_(s^-1^) | proton Larmor frequency (MHz) | longitudinal relaxation rate R_1_(s^-1^) |
| 0.1 *f_BC_* | | 0.2 *f_BC_* | | 0.3 *f_BC_* | | 0.4 *f_BC_* | | 0.5 *f_BC_* | |
| 10.00 | 42.6 ± 1.2 | 10.00 | 33.0 ± 0.5 | 10.00 | 16.9 ± 0.2 | 10.00 | 5.3 ± 0.1 | 10.00 | 8.8 ± 0.2 |
| 8.00 | 43.4 ± 0.8 | 9.00 | 33.6 ± 0.5 | 9.00 | 17.0 ± 0.3 | 9.00 | 5.1 ± 0.2 | 8.00 | 9.4 ± 0.3 |
| 7.00 | 44.6 ± 1.1 | 8.00 | 34.0 ± 0.6 | 8.00 | 17.2 ± 0.3 | 8.00 | 5.2 ± 0.1 | 7.00 | 9.9 ± 0.3 |
| 6.00 | 45.3 ± 1.4 | 7.00 | 33.2 ± 0.6 | 7.00 | 17.0 ± 0.3 | 7.00 | 5.1 ± 0.2 | 6.00 | 10.0 ± 0.3 |
| 5.00 | 48.2 ± 1.4 | 6.00 | 33.4 ± 0.8 | 6.00 | 16.8 ± 0.3 | 6.00 | 5.5 ± 0.2 | 5.00 | 10.2 ± 0.3 |
| 4.00 | 48.1 ± 1.8 | 5.00 | 33.5 ± 0.8 | 5.00 | 17.1 ± 0.4 | 5.00 | 5.6 ± 0.3 | 4.00 | 10.6 ± 0.4 |
| 3.00 | 51.2 ± 1.7 | 4.00 | 35.3 ± 1.3 | 4.00 | 17.0 ± 0.4 | 4.00 | 5.4 ± 0.2 | 3.00 | 11.6 ± 1.6 |
| 3.00 | 50.6 ± 1.3 | 3.00 | 37.4 ± 3.6 | 3.00 | 19.6 ± 1.7 | 3.00 | 5.7 ± 0.2 | 2.00 | 13.3 ± 0.4 |
| 2.00 | 53.0 ± 1.3 | 2.00 | 41 ± 1 | 2.00 | 22.5 ± 0.6 | 2.00 | 6.6 ± 0.2 | 1.50 | 14.2 ± 0.3 |
| 1.00 | 56 ± 1 | 1.00 | 41.2 ± 0.8 | 1.00 | 24.3 ± 0.5 | 1.00 | 7.2 ± 0.2 | 1.00 | 15.0 ± 0.4 |
| 0.60 | 59.4 ± 1.2 | 0.90 | 42.5 ± 0.7 | 0.80 | 25.2 ± 0.6 | 0.90 | 7.2 ± 0.2 | 0.90 | 15.5 ± 0.4 |
| 0.50 | 60.7 ± 5.5 | 0.80 | 43.0 ± 0.8 | 0.60 | 27.3 ± 0.6 | 0.80 | 7.6 ± 0.2 | 0.80 | 15.5 ± 0.4 |
| 0.40 | 62.8 ± 1.3 | 0.50 | 45.8 ± 0.5 | 0.50 | 27.0 ± 0.6 | 0.70 | 7.6 ± 0.2 | 0.70 | 15.8 ± 0.4 |
| 0.30 | 61.9 ± 1.2 | 0.30 | 47.8 ± 0.5 | 0.40 | 27.7 ± 0.6 | 0.50 | 8.2 ± 0.2 | 0.60 | 16.0 ± 0.4 |
| 0.20 | 64.8 ± 1.1 | 0.20 | 48.9 ± 0.7 | 0.30 | 29.1 ± 0.7 | 0.40 | 8.3 ± 0.2 | 0.50 | 17.3 ± 0.4 |
| 0.10 | 70.6 ± 1.4 | 0.10 | 52 ± 1 | 0.20 | 30.3 ± 0.6 | 0.30 | 9.3 ± 0.3 | 0.40 | 17.6 ± 0.4 |
| 0.09 | 70.5 ± 1.1 | 0.09 | 53.1 ± 3.1 | 0.10 | 34.2 ± 0.8 | 0.20 | 10.3 ± 0.2 | 0.30 | 17.8 ± 0.3 |
| 0.07 | 71.6 ± 1.5 | 0.07 | 53.6 ± 0.8 | 0.09 | 36.2 ± 0.7 | 0.10 | 11.3 ± 0.3 | 0.20 | 18.2 ± 0.4 |
| 0.05 | 73.6 ± 1.3 | 0.05 | 55.7 ± 0.8 | 0.07 | 37.3 ± 0.7 | 0.08 | 11.9 ± 0.3 | 0.10 | 19.6 ± 0.3 |
| 0.04 | 75.1 ± 1.5 | 0.04 | 56.6 ± 0.9 | 0.05 | 39.5 ± 0.8 | 0.07 | 12.0 ± 0.2 | 0.09 | 19.4 ± 0.3 |
| 0.03 | 74.9 ± 1.5 | 0.03 | 58.8 ± 0.7 | 0.04 | 40.3 ± 0.7 | 0.05 | 12.9 ± 0.3 | 0.08 | 19.9 ± 0.4 |
| 0.02 | 75.7 ± 1.2 | 0.02 | 62.5 ± 0.7 | 0.03 | 43 ± 1 | 0.04 | 14.0 ± 0.4 | 0.06 | 20.4 ± 0.4 |
| 0.01 | 79.7 ± 1.4 | 0.01 | 64 ± 1 | 0.01 | 48.5 ± 0.8 | 0.03 | 14.8 ± 0.4 | 0.05 | 20.8 ± 0.4 |
|  |  |  |  |  |  | 0.02 | 17.0 ± 0.5 | 0.04 | 21.3 ± 0.5 |
|  |  |  |  |  |  |  |  | 0.03 | 22.8 ± 0.4 |
|  |  |  |  |  |  |  |  | 0.02 | 23.6 ± 0.5 |
|  |  |  |  |  |  |  |  | 0.01 | 25.3 ± 0.4 |
|  |  |  |  |  |  |  |  |  |  |
| proton Larmor frequency (MHz) | longitudinal relaxation rate R_1_(s^-1^) | proton Larmor frequency (MHz) | longitudinal relaxation rate R_1_(s^-1^) | proton Larmor frequency (MHz) | longitudinal relaxation rate R_1_(s^-1^) | proton Larmor frequency (MHz) | longitudinal relaxation rate R_1_(s^-1^) | proton Larmor frequency (MHz) | longitudinal relaxation rate R_1_(s^-1^) |
| 0.6 *f_BC_* | | 0.7 *f_BC_* | | 0.8 *f_BC_* | | 0.9 *f_BC_* | | Biochar | |
| 10.00 | 4.7 ± 0.1 | 10.00 | 3.4 ± 0.1 | 10.00 | 3.1 ± 0.1 | 10.00 | 3.3 ± 0.1 | 10 | 11.5 ± 0.1 |
| 9.00 | 4.7 ± 0.1 | 9.00 | 3.5 ± 0.1 | 9.00 | 3.2 ± 0.1 | 9.00 | 3.4 ± 0.1 | 9 | 11.7 ± 0.1 |
| 8.00 | 4.8 ± 0.1 | 8.00 | 3.5 ± 0.1 | 8.00 | 3.1 ± 0.1 | 8.00 | 3.4 ± 0.1 | 8 | 11.4 ± 0.2 |
| 7.00 | 4.8 ± 0.1 | 7.00 | 3.6 ± 0.1 | 7.00 | 3.2 ± 0.1 | 7.00 | 3.6 ± 0.1 | 7 | 11.3 ± 0.2 |
| 6.00 | 4.8 ± 0.1 | 6.00 | 3.5 ± 0.1 | 6.00 | 3.2 ± 0.1 | 6.00 | 3.6 ± 0.1 | 6 | 11.4 ± 0.2 |
| 5.00 | 4.9 ± 0.1 | 5.00 | 3.8 ± 0.1 | 5.00 | 3.4 ± 0.1 | 5.00 | 3.6 ± 0.1 | 5 | 11.6 ± 0.3 |
| 4.00 | 5.2 ± 0.1 | 4.00 | 3.8 ± 0.1 | 4.00 | 3.5 ± 0.1 | 4.00 | 3.7 ± 0.1 | 4 | 11.5 ± 0.3 |
| 3.00 | 5.2 ± 0.1 | 3.00 | 3.9 ± 0.1 | 3.00 | 3.5 ± 0.1 | 3.00 | 4.2 ± 0.1 | 3 | 11.9 ± 0.3 |
| 2.00 | 5.6 ± 0.1 | 3.00 | 3.8 ± 0.1 | 2.00 | 3.9 ± 0.1 | 2.00 | 4.3 ± 0.1 | 2 | 12.4 ± 0.2 |
| 1.00 | 5.9 ± 0.1 | 2.00 | 4.2 ± 0.1 | 1.00 | 4.1 ± 0.1 | 1.50 | 4.5 ± 0.1 | 1.5 | 13.1 ± 0.2 |
| 0.80 | 6.0 ± 0.1 | 1.00 | 4.7 ± 0.1 | 0.90 | 4.2 ± 0.1 | 1.00 | 4.7 ± 0.1 | 1 | 13.2 ± 0.2 |
| 0.60 | 6.4 ± 0.1 | 0.80 | 4.8 ± 0.1 | 0.70 | 4.4 ± 0.1 | 0.90 | 4.7 ± 0.1 | 0.8 | 13.3 ± 0.2 |
| 0.50 | 6.5 ± 0.1 | 0.60 | 5.0 ± 0.1 | 0.50 | 4.6 ± 0.1 | 0.70 | 4.8 ± 0.1 | 0.6 | 13.4 ± 0.2 |
| 0.40 | 6.7 ± 0.1 | 0.50 | 5.1 ± 0.1 | 0.40 | 4.9 ± 0.1 | 0.60 | 4.9 ± 0.1 | 0.4 | 13.7 ± 0.2 |
| 0.30 | 7.0 ± 0.1 | 0.40 | 5.4 ± 0.1 | 0.30 | 5.1 ± 0.1 | 0.50 | 5.2 ± 0.1 | 0.3 | 14.1 ± 0.2 |
| 0.20 | 7.4 ± 0.1 | 0.30 | 5.6 ± 0.1 | 0.20 | 5.6 ± 0.1 | 0.40 | 5.3 ± 0.1 | 0.2 | 14.5 ± 0.2 |
| 0.10 | 8.2 ± 0.1 | 0.20 | 6.4 ± 0.1 | 0.10 | 6.4 ± 0.1 | 0.30 | 5.7 ± 0.1 | 0.15 | 14.4 ± 0.2 |
| 0.08 | 8.7 ± 0.2 | 0.10 | 7.3 ± 0.1 | 0.09 | 6.7 ± 0.1 | 0.20 | 6.2 ± 0.1 | 0.1 | 14.9 ± 0.2 |
| 0.06 | 9.2 ± 0.1 | 0.09 | 7.9 ± 0.1 | 0.07 | 7.2 ± 0.1 | 0.10 | 7.2 ± 0.1 | 0.08 | 15.3 ± 0.2 |
| 0.05 | 9.4 ± 0.1 | 0.07 | 8.3 ± 0.1 | 0.05 | 7.9 ± 0.1 | 0.09 | 7.3 ± 0.1 | 0.06 | 15.7 ± 0.2 |
| 0.03 | 10.8 ± 0.1 | 0.06 | 8.4 ± 0.1 | 0.04 | 8.8 ± 0.1 | 0.07 | 7.8 ± 0.1 | 0.04 | 16.3 ± 0.2 |
| 0.02 | 11.5 ± 0.1 | 0.05 | 8.9 ± 0.1 | 0.03 | 9.2 ± 0.1 | 0.06 | 8.1 ± 0.1 | 0.03 | 17.1 ± 0.2 |
| 0.01 | 11.9 ± 0.1 | 0.04 | 9.5 ± 0.2 | 0.02 | 9.8 ± 0.1 | 0.05 | 8.2 ± 0.1 | 0.02 | 17.2 ± 0.2 |
|  |  | 0.03 | 9.8 ± 0.2 | 0.01 | 10.3 ± 0.1 | 0.04 | 8.9 ± 0.1 | 0.015 | 17.1 ± 0.1 |
|  |  | 0.02 | 10.5 ± 0.2 |  |  | 0.03 | 9.3 ± 0.1 | 0.01 | 17.3 ± 0.3 |
|  |  | 0.01 | 11.9 ± 0.2 |  |  | 0.02 | 9.8 ± 0.1 |  |  |
|  |  |  |  |  |  | 0.01 | 10.8 ± 0.1 |  |  |


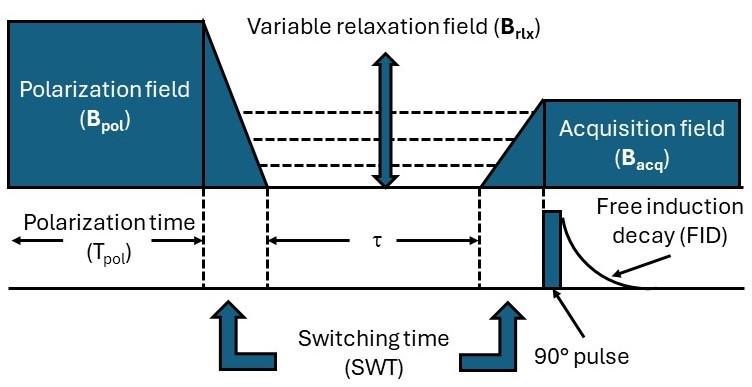


### **Figure S1.**

Pulse sequence to acquire a fast field cycling NMR relaxometry experiment. The reader is addressed to the main text for the details about the sequence.

## **REFERENCES**

Bortolotti, V., Conte, P., Landi, G., Lo Meo, P., Nagmutdinova, A., Spinelli, G.V., Zama, F., 2024. Robust Algorithms for the Analysis of Fast-Field-Cycling Nuclear Magnetic Resonance Dispersion Curves. Computers 13, 129. https://doi.org/10.3390/computers13060129

Conte, P., 2021. Applications of Fast Field Cycling NMR Relaxometry, in: Webb, G.A. (Ed.), Annual Reports on NMR Spectroscopy. Academic Press - Elsevier, London, UK, pp. 141–188. https://doi.org/10.1016/bs.arnmr.2021.05.001

Conte, P., Lo Meo, P., 2020. Nuclear Magnetic Resonance with Fast Field-Cycling Setup: A Valid Tool for Soil Quality Investigation. Agronomy 10, 1040. https://doi.org/10.3390/agronomy10071040

Kimmich, R., Anoardo, E., 2004. Field-cycling NMR relaxometry. Prog. Nucl. Magn. Reson. Spectrosc. 44, 257–320. https://doi.org/10.1016/j.pnmrs.2004.03.002

Landi, G., Spinelli, G. V., Zama, F., Martino, D.C., Conte, P., Lo Meo, P., Bortolotti, V., 2023. An automatic L1-based regularization method for the analysis of FFC dispersion profiles with quadrupolar peaks. Appl. Math. Comput. 444, 1–16. https://doi.org/10.1016/j.amc.2022.127809

Lo Meo, P., Terranova, S., Di Vincenzo, A., Chillura Martino, D., Conte, P., 2021. Heuristic Algorithm for the Analysis of Fast Field Cycling (FFC) NMR Dispersion Curves. Anal. Chem. 93, 8553–8558. https://doi.org/10.1021/acs.analchem.1c01264
